# Supplementary material for: Super-spreading events initiated the exponential growth phase of COVID-19 with ℛ0 higher than initially estimated
Source: R Soc Open Sci. 2020 Sep 23;7(9):200786. doi: 10.1098/rsos.200786 (PMC7540800; doi:10.1098/rsos.200786)
Supplement: Electronic Supplementary Material [file rsos200786supp1.pdf]

ESM

rsos.royalsocietypublishing.org R. Soc. open sci. 000000

# Super-spreading events initiated the exponential growth phase of COVID-19 with $R_0$ higher than initially estimated

**Marek Kochańczyk, Frederic Grabowski, & Tomasz Lipniacki**

This material features an article

published in the *Royal Society Open Science* 2020.

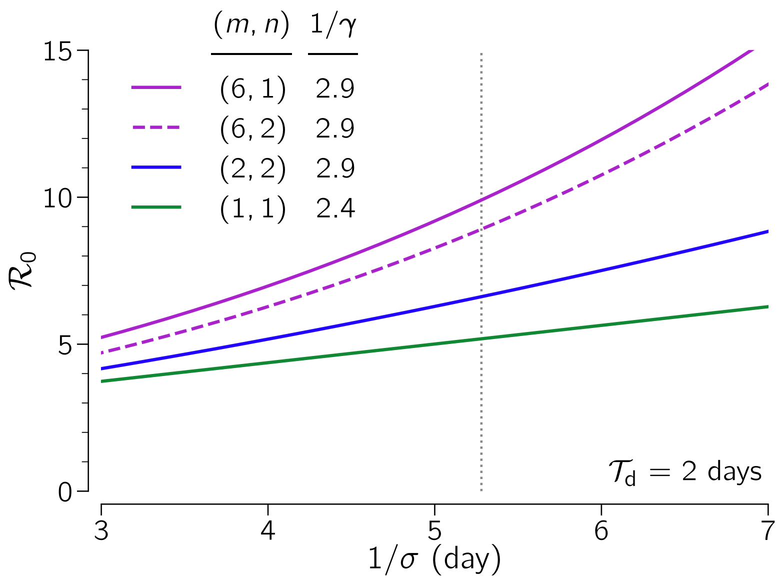


**Figure S1.** **Basic reproduction number, $\mathcal{R}_0$, *vs.* mean latent period, $1/\sigma$.** The SEIR model parameters are given in the legend; default $1/\sigma$ = 5.28 days is marked with a dotted vertical line. The assumed doubling time $\mathcal{T}_d$ is 2 days.

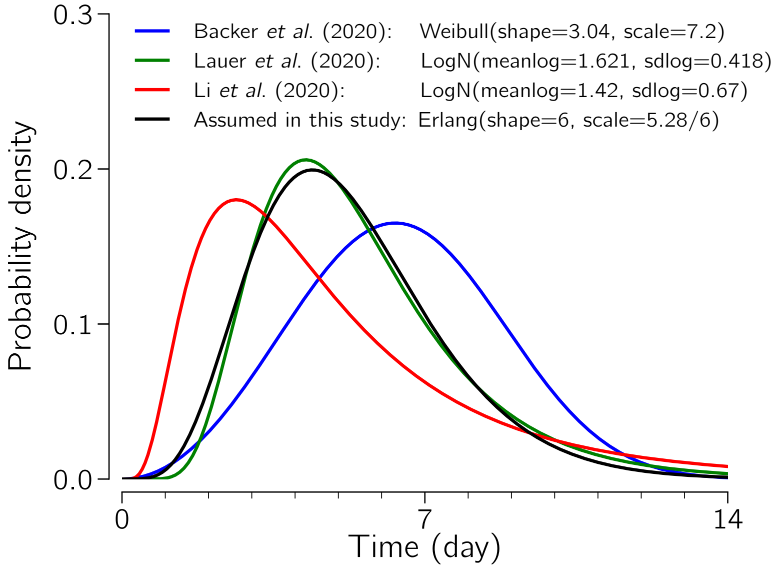


**Figure S2.** **Incubation period distributions**. Three probability density functions taken from literature and the density assumed in this study are shown together.

Backer *et al.* found that incubation period follows Weibull distribution with mean equal 6.4 days and standard deviation equal 2.3 days. This gives shape parameter 3.04 and scale parameter 7.2 days [1]. Lauer *et al.* found that incubation period follows log-normal distribution with meanlog equal 1.621 and sdlog equal 0.418. [2]. Based on very limited data, Li *et al.* proposed that incubation period follows log-normal distribution with mean equal 5.2 days and 95th percentile of the distribution at 12.5 days which corresponds to meanlog equal 1.42 and sdlog equal 0.67 [3].

## References

1. Backer JA, Klinkenberg D, Wallinga J. 2020 Incubation period of 2019 novel coronavirus (2019-nCoV) infections among travellers from Wuhan, China, 20–28 January 2020. *Eurosurveillance* **25**. (doi:10.2807/1560-7917.ES.2020.25.5.2000062).
2. Lauer SA, Grantz KH, Bi Q, Jones FK, Zheng Q, Meredith H, Azman AS, Reich NG, Lessler J. 2020 The incubation period of 2019-nCoV from publicly reported confirmed cases: Estimation and application. *medRxiv*. (doi:10.1101/2020.02.02.20020016).
3. Li Q, Guan X, Wu P, Wang X, Zhou L, Tong Y, Ren R, Leung KS, Lau EH, Wong JY, Xing X, Xiang N, Wu Y, Li C, Chen Q, Li D, Liu T, Zhao J, Liu M, Tu W, Chen C, Jin L, Yang R, Wang Q, Zhou S, Wang R, Liu H, Luo Y, Liu Y, Shao G, Li H, Tao Z, Yang Y, Deng Z, Liu B, Ma Z, Zhang Y, Shi G, Lam TT, Wu JT, Gao GF, Cowling BJ, Yang B, Leung GM, Feng Z. 2020 Early Transmission Dynamics in Wuhan, China, of Novel Coronavirus–Infected Pneumonia. *New England Journal of Medicine* **382**, 1199–1207. (doi: 10.1056/NEJMoa2001316).

**Listing S1. BioNetGen language (BNGL)-encoded SEIR-type model of epi demic spread in the presence of super-spreaders.** Parameters of the model are set so that the proportion of superspreaders in the population is set according to their infectiousness (relative w.r.t. to "normal" individuals) so that both groups, superspreaders and "normal" individuals, have identical total effective infectiousness. Also, contact rate is normalized so that the number of infecter "normal" individuals does not depend on the current relative infectiousness of superspreaders. The file contains both the model definition and, at the bottom, simu- lation protocol that runs a simulation with or without superspreaders. By changing `method=>...`, one can run either a deterministic simulation (`method=>ode`), using numerical integration of a resulting system of ODES, or an exact stochastic simulation (`method=>ssa`) of a corresponding Markov chain, performed according the Gillespie algorithm. To simulate model dynamics, please download and install BioNetGen (from http://bionetgen.org). For the sake of convenience, you may use BioNetGen within RuleBender (http://www.rulebender.org). The model was developed in BioNetGen version 2.4.0 (in hope that it will be also compatible with future BioNetGen releases).

```
begin model

  begin parameters
    super_fraction      1/(100 - 1)
    super_strength      1/super_fraction
  # ^--- To simulate hyperspreaders, multiply super_strength by 2.

    normal_prob         1/(1 + super_fraction)                # => 99%
    super_prob          super_fraction/(1 + super_fraction)   # =>  1%
    infected_0_normal   1   # |_ initial condition
    infected_0_super    0   # |
    z          (1 + super_fraction*super_strength)/(1 + super_fraction)

    beta     3.41478/z      # contact rate [1/day]   # => Td == 2 days
    gamma    1/2.9          # removal rate [1/day]
    tau      5.28           # average incubation period  [day]
    k        6              # no. exposed states
  # ^--- This value scales rates and does not affect model structure.
  end parameters

  begin seed species
    E1_super()      0
    E1_normal()     0
    E2_super()      0
    E2_normal()     0
    E3_super()      0
    E3_normal()     0
    E4_super()      0
    E4_normal()     0
    E5_super()      0
    E5_normal()     0
    E6_super()      0
    E6_normal()     0
    I_super()       infected_0_super
    I_normal()      infected_0_normal
    R_super()       0
    R_normal()      0
    event_normal()  0
    event_super()   0
  end seed species
```

```
  begin observables
    # -- individuals in SEIR compartments
    Molecules E1_super  E1_super()
    Molecules E1_normal E1_normal()
    Molecules E2_super  E2_super()
    Molecules E2_normal E2_normal()
    Molecules E3_super  E3_super()
    Molecules E3_normal E3_normal()
    Molecules E4_super  E4_super()
    Molecules E4_normal E4_normal()
    Molecules E5_super  E5_super()
    Molecules E5_normal E5_normal()
    Molecules E6_super  E6_super()
    Molecules E6_normal E6_normal()
    Molecules I_super   I_super()
    Molecules I_normal  I_normal()
    Molecules R_super   R_super()
    Molecules R_normal  R_normal()

    # -- transmission event counters
    Molecules event_normal event_normal()
    Molecules event_super  event_super()
  end observables

  begin reaction rules
    I_normal() -> I_normal() + E1_normal() + event_normal() normal_prob*beta
    I_normal() -> I_normal() + E1_super()  + event_normal() super_prob*beta
    I_super()  -> I_super()  + E1_normal() + event_super() normal_prob*beta*super_strength
    I_super()  -> I_super()  + E1_super()  + event_super() super_prob*beta*super_strength
    E1_super()  -> E2_super()   k/tau
    E1_normal() -> E2_normal()  k/tau
    E2_super()  -> E3_super()   k/tau
    E2_normal() -> E3_normal()  k/tau
    E3_super()  -> E4_super()   k/tau
    E3_normal() -> E4_normal()  k/tau
    E4_super()  -> E5_super()   k/tau
    E4_normal() -> E5_normal()  k/tau
    E5_super()  -> E6_super()   k/tau
    E5_normal() -> E6_normal()  k/tau
    E6_super()  -> I_super()    k/tau
    E6_normal() -> I_normal()   k/tau
    I_super()   -> R_super()    gamma
    I_normal()  -> R_normal()   gamma
  end reaction rules

end model


generate_network({overwrite=>1});
simulate({method=>"ssa", suffix=>"ssa", t_end=>60, n_steps=>600, \
         stop_if=>"R_normal() + R_super() > 5000000"})
```
